# Identification of kinase inhibitors as potential host-directed therapies for intracellular bacteria

**DOI:** 10.1038/s41598-024-68102-6

**Published:** 2024-07-26

**Authors:** Robin H. G. A. van den Biggelaar, Kimberley V. Walburg, Susan J. F. van den Eeden, Cassandra L. R. van Doorn, Eugenia Meiler, Alex S. de Ries, M. Chiara Fusco, Annemarie H. Meijer, Tom H. M. Ottenhoff, Anno Saris

**Affiliations:** 1 Leiden University Center for Infectious Diseases, Leiden University Medical Center, Leiden, The Netherlands; 2 Institute of Biology Leiden, Leiden University, Leiden, The Netherlands; 3 Global Health Medicines R&D, GlaxoSmithKline, Tres Cantos, Spain

**Keywords:** Antimicrobials, Tuberculosis, Cellular microbiology, Bacterial infection

## Abstract

The emergence of antimicrobial resistance has created an urgent need for alternative treatments against bacterial pathogens. Here, we investigated kinase inhibitors as potential host-directed therapies (HDTs) against intracellular bacteria, specifically *Salmonella* Typhimurium (*Stm*) and *Mycobacterium tuberculosis* (*Mtb*). We screened 827 ATP-competitive kinase inhibitors with known target profiles from two Published Kinase Inhibitor Sets (PKIS1 and PKIS2) using intracellular infection models for *Stm* and *Mtb*, based on human cell lines and primary macrophages. Additionally, the in vivo safety and efficacy of the compounds were assessed using zebrafish embryo infection models. Our screen identified 11 hit compounds for *Stm* and 17 hit compounds for *Mtb* that were effective against intracellular bacteria and non-toxic for host cells. Further experiments were conducted to prioritize *Stm* hit compounds that were able to clear the intracellular infection in primary human macrophages. From these, two structurally related *Stm* hit compounds, GSK1379738A and GSK1379760A, exhibited significant activity against *Stm* in infected zebrafish embryos. In addition, we identified compounds that were active against intracellular *Mtb*, including morpholino-imidazo/triazolo-pyrimidinones that target PIK3CB, as well as 2-aminobenzimidazoles targeting ABL1. Overall, this study provided insights into kinase targets acting at the host–pathogen interface and identified several kinase inhibitors as potential HDTs.

## Introduction

Infectious diseases caused by intracellular bacteria such as *Salmonella* species and *Mycobacterium tuberculosis* (*Mtb*) significantly impact global health. There are an estimated 100 million annual cases of *Salmonella*-related diseases worldwide, resulting in approximately 250,000 deaths^1,2^. In addition, *Mtb* is estimated to be latently present in almost a quarter of the world's population, with around 10 million new cases of tuberculosis (TB) each year, and around 1.5 million deaths^2–4^. A significant challenge in treating these bacterial infections is the rise of antimicrobial resistance, which highlights the need for alternative therapeutic approaches^1,4,5^.

As both *Salmonella* species and *Mtb* are facultative intracellular pathogens, host-directed therapy (HDT) may provide a promising alternative for treatment. HDT aims to target the host response against infection rather than the pathogen itself and may be used as an adjunctive or alternative treatment to classical antibiotics. Importantly, HDTs have the potential to target antibiotic resistant strains. Due to their involvement in many host–pathogen interaction mechanisms, kinase inhibitors have previously gained attention as HDT candidates against intracellular bacteria. Both *Salmonella enterica* serovar Typhimurium (*Stm*) and *Mtb* activate the phosphoinositide 3-kinase (PI3K)/AKT signaling pathway through their virulence factors to control intracellular trafficking, prevent phagosome-lysosome fusion and stimulate their survival in the host cell^6–10^. Consequently, chemical inhibition (e.g. H-89 and AR-12) or genetic inhibition of kinases that are part of this signaling pathway alleviate this control and impair bacterial growth^6–10^. Furthermore, chemical or genetic inhibition of several receptor tyrosine kinases stimulates host cell control of intracellular *Mtb*^6,7^. In particular, gefitinib and other inhibitors of EGFR have been well studied and were found to reduce *Mtb* and *Stm* intracellular burden, possibly by enhancing lysosomal function and inducing metabolic changes^7–10^. In addition, inhibition of ABL1 by GNF-2 or imatinib consistently reduces intracellular *Mtb* burden, most likely by overcoming pathogen-driven suppression of lysosomal acidification^6–8,11–15^. Currently, the ABL1 inhibitor imatinib is being tested in clinical trials as adjunctive therapy together with an antibiotic regimen of rifabutin and isoniazid against drug-sensitive TB^16^.

In this study, we explored the potential of well-characterized kinase inhibitors as candidates for HDT by screening 827 ATP-competitive kinase inhibitors of two Published Kinase Inhibitor Sets, PKIS1 and PKIS2, in intracellular infection models for *Stm* and *Mtb*^17,18^. We performed flow cytometry-based screens to identify HDTs, using previously validated HeLa and MelJuSo infection models that, unlike monocytic cell lines, do not require phorbol 12-myristate 13-acetate-mediated differentiation, to avoid altering any host kinase signaling networks prior to the experiment^6,19^. Next, we examined potential HDT targets by combing data from this chemical kinase inhibition screen with a genetic kinase inhibition screen^6^. Finally, we evaluated promising compounds in vitro in primary human macrophages and in vivo in zebrafish embryo models. The findings of this study resulted in the identification of novel HDTs as well as HDT targets critical for host–pathogen-interactions, which will help to overcome the challenges posed by emerging antibiotic resistance.

## Results

### Screening the PKIS library against intracellular *Stm* and *Mtb* identifies novel kinase inhibitors for host-directed therapy

In this study, a library of 827 PKIS kinase inhibitors was consecutively subjected to two flow cytometry-based screens to identify novel host-directed therapeutics with antimicrobial activity against intracellular *Stm* and *Mtb*. The results of the screen with *Stm*-DsRed-infected HeLa cells showed that two populations of infected cells could be discerned by flow cytometry, namely DsRed-dim and DsRed-bright (Supplementary Fig. S1a). For *Mtb*, only one DsRed+ population was observed (Supplementary Fig. S1b). As previously reported, the positive control H-89, a PKA and AKT/PKB inhibitor known to reduce intracellular *Stm* bacterial burden, was found to diminish mainly the DsRed-bright population^6^. Since we observed the same phenomenon for several PKIS compounds, we set out to determine the contribution of the DsRed-dim and DsRed-bright populations of *Stm*-infected HeLa cells to the actual bacterial burden in order to choose the best outcome parameter. First, the presence of both populations was determined 1 h post infection and the DsRed-bright population was absent at this early timepoint (Supplementary Fig. S2a). Next, after overnight incubation the DsRed-dim and DsRed-bright *Stm*-infected HeLa populations were sorted by FACS and lysed to determine the average colony-forming units (CFUs)/cell. DsRed-bright cells contained 142 times more viable bacteria than DsRed-dim cells (Supplementary Figs. S2b, c). Combined, these results show that the DsRed-bright population comprises HeLa cells with replicating *Stm*, thus being most relevant as outcome parameter. The primary screen conducted with *Stm*-infected HeLa cells identified 82 inhibitors resulting in smaller DsRed-bright populations (z-score DsRed-bright <  − 2; Supplementary Fig. S1c and e and Supplementary Table S1), of which 56 were not cytotoxic as determined by cellular counts by flow cytometry (z-score cell count >  − 3; Supplementary Fig. S1e). The screen conducted with *Mtb*-infected MelJuSo cells identified 66 inhibitors (z-score DsRed+  <  − 2; Supplementary Fig. S1d and e), of which 44 compounds did not result in cytotoxicity (z-score cell count >  − 3; Supplementary Fig. S1e). From the 56 and 44 inhibitors of intracellular *Stm* and *Mtb*, respectively, 7 compounds were active against both pathogens.

**Figure 1:**
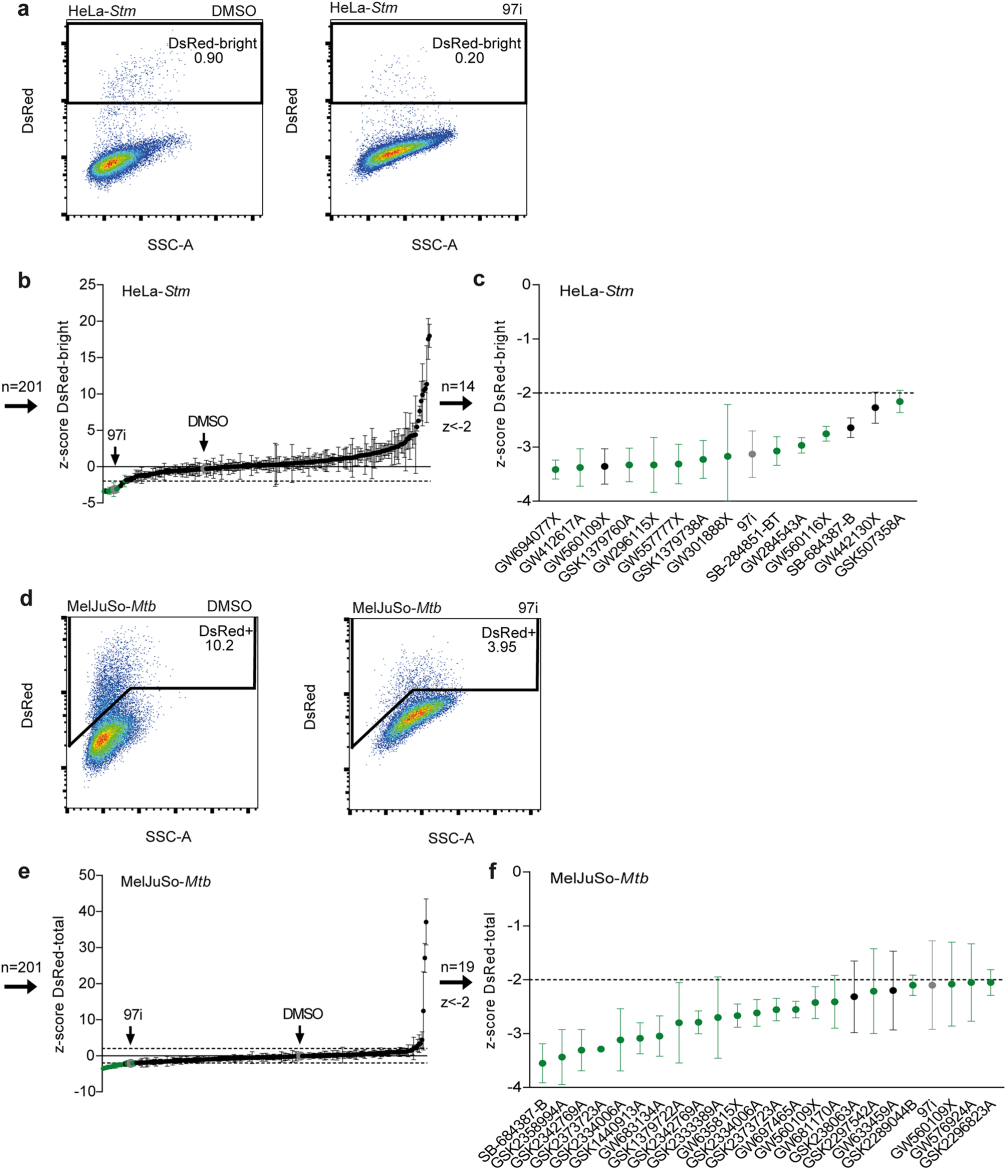
Identification of PKIS compounds inhibiting intracellular growth of *Stm* and *Mtb*. (**a**) Gating strategy for DsRed-bright *Stm*-infected HeLa cells to determine the percentage of infected cells. The negative control treated with DMSO and positive control treated with 97i are depicted in grey. (**b**) Rescreen of 201 PKIS compounds, 30 of which appear twice, to assess their impact on *Stm* bacterial burden, expressed as average z-scores of the DsRed-bright population. (**c**) Hit compounds with z-scores < − 2. PKIS compounds that reduced bacterial burden in both the primary screen and rescreen are depicted in green, while black dots represent compounds that were only effective in one of the two screens. Controls are depicted in grey. (**d**) Gating strategy for DsRed+ *Mtb*-infected MelJuSo cells. (**e**) Re-screen on *Mtb*-infected MelJuSo cells. (**f**) Hit compounds with z-scores < − 2 for *Mtb*. The screens were performed with three technical replicates, and error bars show standard deviations.

Rescreens were performed in order to select for compounds that consistently reduced the intracellular bacterial burden. For the rescreen a smaller set of PKIS compounds was used, comprising 201 compounds that were non-cytotoxic in the primary screen and found to be active, affecting the bacterial burden either positively or negatively (z-scores <  − 2 or > 2; Supplementary Fig. S1e). For *Stm*, 14 inhibitory hit compounds were identified in the rescreen (z-score <  − 2), 11 of which also inhibited *Stm* in the primary screen and these were selected for further experiments (Figs. 1a–c). For *Mtb*, 19 unique compounds were found to reduce the intracellular burden in the rescreen (z-score <  − 2), 17 of which also inhibited *Mtb* in the primary screen. (Figs. 1d–f). As one *Mtb* compound (*i.e.*, GSK2289044B) could not be included in subsequent experiments because additional quantities were not available, 16 *Mtb*-effective compounds were selected for further experiments. In line with the cytotoxicity data from the primary screen, none of the *Stm* and *Mtb* hit compounds significantly affected the cell count (Supplementary Table S1). Ultimately, the screen resulted in 11 hit compounds against intracellular *Stm* and 16 hit compounds against intracellular *Mtb* that were further evaluated.

### 2-anilino-4-pyrrolidinopyrimidines targeting JAK2 and AAK1, and 4-anilinoquinolines targeting MAP2K5, RIPK2 and RSK4 are active against intracellular *Stm*

**Figure 2:**
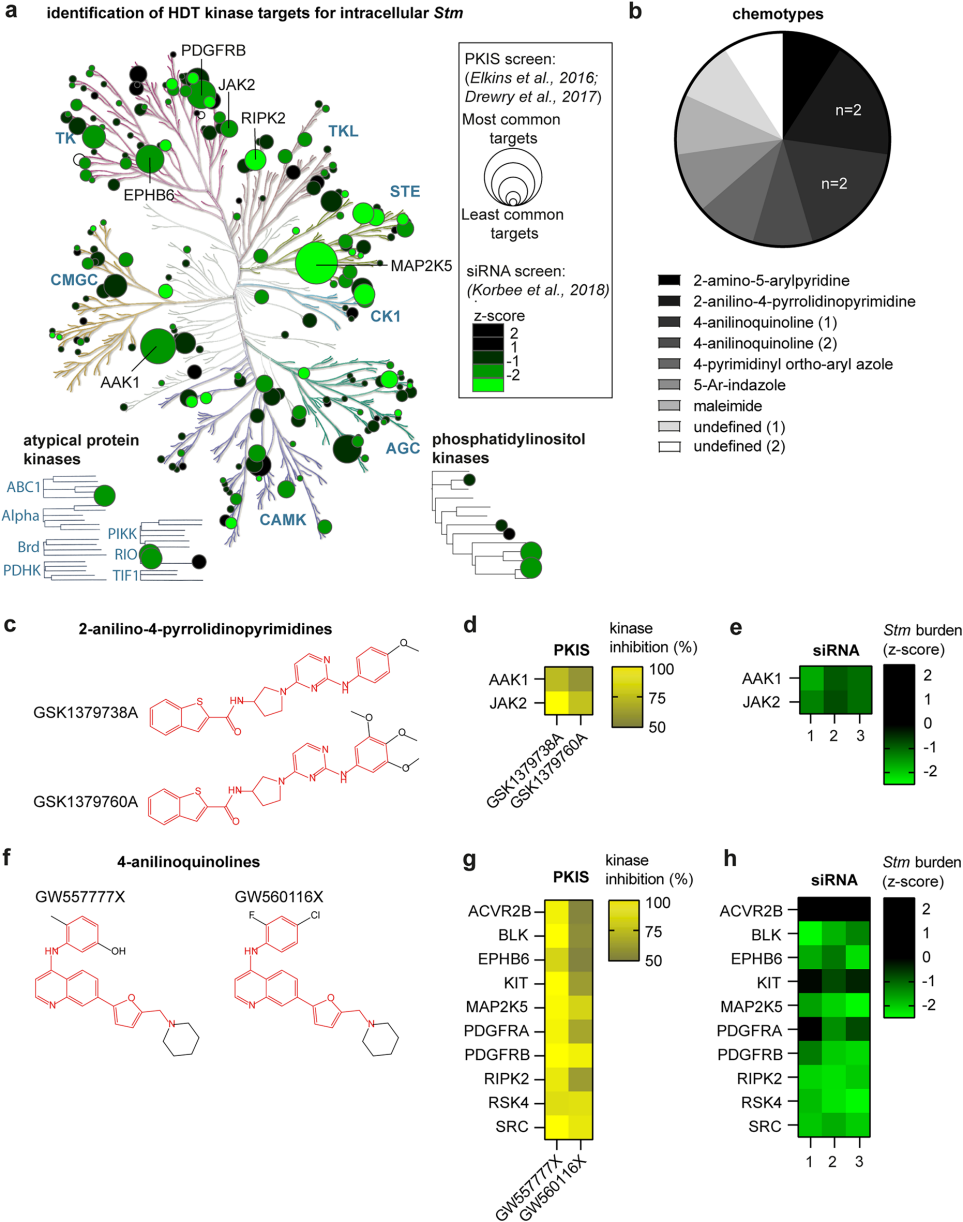
Identification of HDT targets against intracellular *Stm* through combined chemical and genetic inhibition of host kinases. (**a**) Each circle in the phylogenetic tree depicts host kinases that are relevant for HDT against intracellular *Stm* by combining data from chemical^19,20^ and genetic^6^ kinase inhibition screens. The effect of chemical inhibition of kinase targets is represented by circle size, which corresponds to the number of *Stm* hit compounds that inhibit a kinase by > 50%^51^. The effect of genetic inhibition of kinase targets, using a siRNA knockdown screen of host kinases in *Stm*-infected HeLa cells, is shown by color. The most relevant targets for *Stm* survival are shown in green, while likely off-targets are shown in black. White circles represent targets for which genetic inhibition data were not available. (**b**) All 11 *Stm* hit compounds were clustered according to chemotype and chemical similarity, using the Tanimoto coefficient as a similarity measure with cut-off value of 0.5^51^. (**c**) Chemical structures of the 2-anilino-4-pyrrolidinopyrimidine *Stm* hit compounds GSK1379738A and GSK1379760A. (**d**) The level of inhibition of kinase targets shared by GSK1379760A and GSK1379738A at 1 μM. (**e**) Effect of knockdown of JAK2 and AAK1 on the bacterial burden of *Stm*-infected HeLa cells. (**f**) Chemical structures of the 4-anilinoquinoline *Stm* hit compounds GW557777X and GW560116X. (**g**) The level of inhibition of kinase targets shared by GW557777X and GW560116X at 1 μM. (**h**) Effect of knockdown of the same kinase targets on the bacterial burden of *Stm*-infected HeLa cells.

Based on the published biochemical kinase inhibition profiles of the PKIS compounds at 1 µM, we determined which host kinases are likely important for host–pathogen interactions of *Stm*-infected cells^17,18^. The *Stm* hit compounds comprised 2 PKIS1 compounds (GW296115X and GW301888X) and 9 PKIS2 compounds that have been assessed for their activity at 1 µM concentration against 203 and 392 wildtype kinases, respectively. For each kinase, compounds showing > 50% kinase inhibition were counted to identify kinases important for intracellular *Stm* survival. Overall, the most commonly targeted kinases by these compounds were MAP2K5 (6/9), PDGFRB (5/11), EPHB6 (4/9) and AAK1 (5/9) (Fig. 2a). To ensure that the kinases were not simply common targets in the PKIS library (i.e., association bias), we compared the degree of inhibition of identified kinases between hit compounds and non-hit compounds. The hit compounds exhibited greater inhibition in all identified targets, although this difference was only statistically significant for AAK1 (Supplementary Fig. S3a). In addition, we determined the individual contribution of kinase targets to intracellular *Stm* survival in infected HeLa cells based on the results of a kinome siRNA knockdown screen used^6^. Knockdown of the identified kinase targets also resulted in a lower intracellular *Stm* burden in infected HeLa cells, which was strongest for MAP2K5 (z = − 2.1). *Stm* hit compounds were structurally diverse, encompassing eight different chemotypes and comprising two pairs of structurally similar compounds (Fig. 2b). The 2-anilino-4-pyrrolidinopyrimidines GSK1379738A and GSK1379760A only differed in their 2-anilino group, with GSK1379760A possessing two additional 3- and 5- methoxyl groups (Fig. 2c). Despite their structural overlap, these compounds show limited overlap in their kinase targets, with the exception of JAK2 and AAK1 (Figs. 2c and d). Individual knockdown of JAK2 (z = − 1.0) and AAK1 (z = − 1.2) resulted in a modest decrease in the *Stm* intracellular burden (Fig. 2e). The 4-anilino-quinolines comprised GW557777X and GW560116X, which only differed in their 4-anilino group, with GW557777X possessing 2-methyl and 5-hydroxyl groups and GW560116X possessing 2-fluorine and 4-chlorine groups (Fig. 2f). The compounds have many kinase targets in common including ACVR2B, BLK, EPHB6, KIT, MAP2K5, PDGFRA, PDGFRB, RIPK2, RSK4 and SRC (Fig. 2g). Compound GW284543A, a structurally different 4-anilinoquinoline, also targets BLK, EPHB6, RIPK2 and MAP2K5. Individual knockdown of MAP2K5 (z = − 2.1), RIPK2 (z = − 2.1) and RSK4 (z = − 2.2) reduced the *Stm* burden of infected HeLa cells, identifying these kinases as important targets (Fig. 2h). Finally, compound GSK507358A, an inhibitor of all AKT isoforms, may act in a similar manner as H-89 and 97i by targeting AKT1^14,20,21^. Of note, two similar AKT-targeting compounds, GSK682037B and GSK562689A, also strongly reduced the *Stm*-bright population, but were excluded due to cytotoxicity (Supplementary Table S1). In summary, we found MAP2K5, RIPK2 and RSK4 as host kinases that are important for the host–pathogen interactions of *Stm*-infected cells, while inhibition of AAK1 and JAK2 separately contributes, but is not sufficient, to reduce intracellular *Stm* burden.

### Morpholino-imidazo/triazolo-pyrimidinones targeting PIK3CB and 2-aminobenzimidazoles targeting ABL1 are effective against intracellular *Mtb*

**Figure 3:**
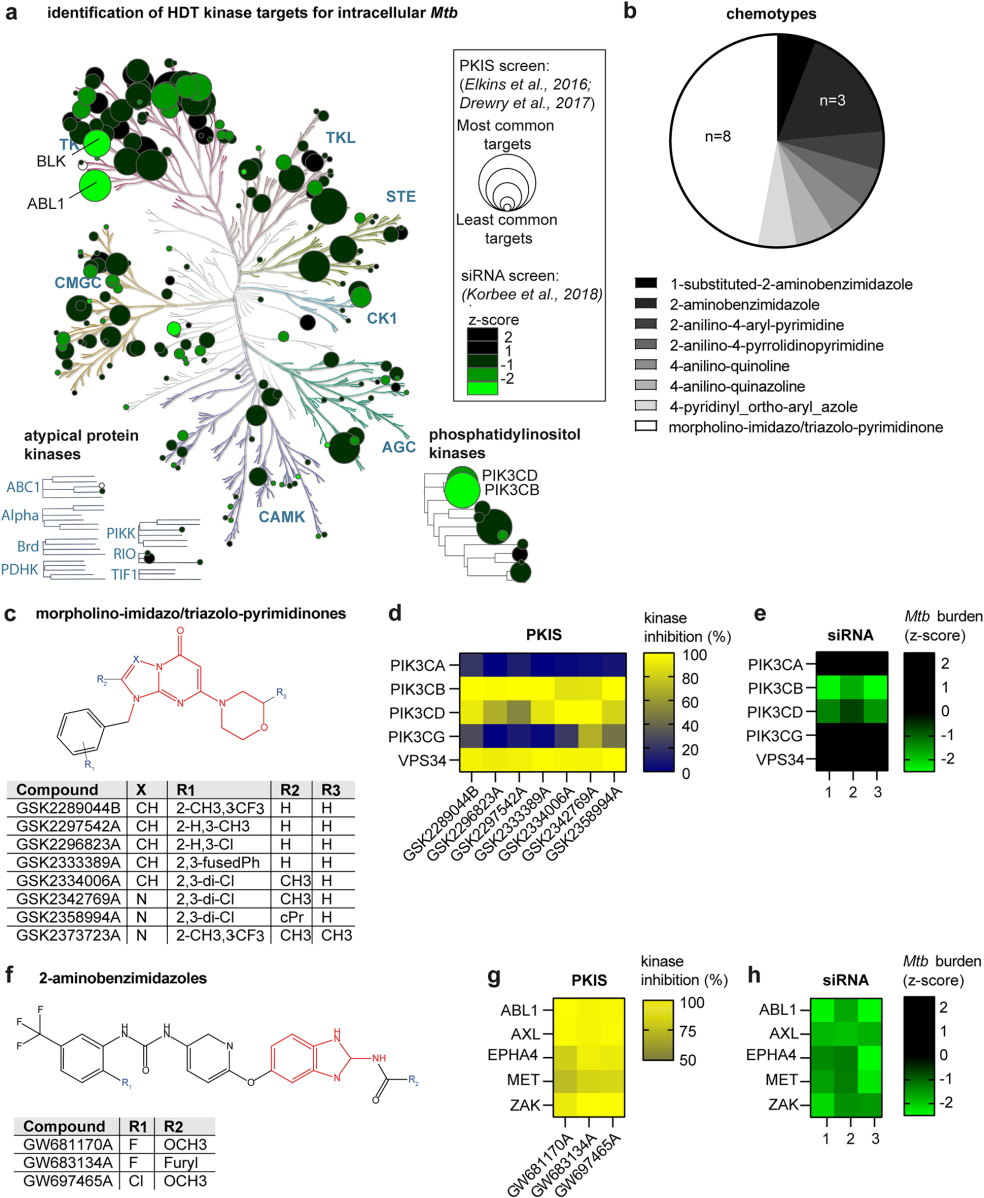
Identification of HDT targets against intracellular *Mtb* through combined chemical and genetic inhibition of host kinases. (**a**) Each circle in the phylogenetic tree depicts host kinases that are relevant for HDT against intracellular *Mtb* by combining data from chemical^19,20^ and genetic^6^ kinase inhibition screens. The effect of chemical inhibition of kinase targets is represented by circle size, which corresponds to the number of *Mtb* hit compounds that inhibit a kinase by > 50%^51^. The effect of genetic inhibition of kinase targets, using a siRNA knockdown screen of host kinases in *Mtb*-infected MelJuSo cells, is shown by color with the most relevant targets in *Mtb* survival shown in green in green for relevant targets involved in *Mtb* survival, while likely off-targets are shown in black. White circles represent targets for which genetic inhibition was not available. (**b**) All 17 *Mtb* hit compounds were clustered according to chemotype and chemical similarity, using the Tanimoto coefficient as a similarity measure with cut-off value of 0.5^51^. (**c**) Chemical structures of *Mtb* hit compounds belonging to the chemotype of morpholino-imidazo/triazolo-pyrimidinones. (**d**) Inhibition of phosphatidyl inositol 3-kinases by morpholino-imidazo/triazolo-pyrimidinone hit compounds. (**e**) Effect of genetic inhibition of phosphatidyl inositol 3-kinases on the bacterial burden of *Mtb*-infected MelJuSo cells. (**f**) Chemical structures of *Mtb* hit compounds belonging to the chemotype of 2-aminobenzimidazoles. (**g**) Inhibition of selected kinases by 2-aminobenzimidazole *Mtb* hit compounds. Only the five shared kinases that gave the strongest effect on intracellular *Mtb* upon knockdown are shown (see Supplementary Figure 4﻿ for the whole list of 51 shared targets). (**h**) Effect of knockdown of the same kinase targets on the bacterial burden of *Mtb*-infected MelJuSo cells.

The *Mtb* hit compounds comprised 1 PKIS1 compound (i.e., GW576924A), 15 PKIS2 compounds and 1 compound that is part of both sets (i.e., GW683134A). Commonly targeted kinases by these compounds were ABL1 (7/17), EPHA1 (6/15), EPHB6 (6/15), RET (9/17), MAP3K19 (7/15), MAP2K5 (6/15), CRIK (6/15), PIK3CB (8/15), PIK3CD (7/17) and VPS34 (8/15) (Fig. 3a). Again, we compared the degree of inhibition of the identified kinase targets between the hit compounds and other compounds of the PKIS library and found that RET, PIK3CB, PIK3CD and VPS34 were inhibited significantly more by hit compounds (Supplementary Fig. S3b). Furthermore, data from a kinome siRNA knockdown screen show that individual knockdown of ABL1 (z = − 2.2), PIK3CB (z = − 2.3), and to a lesser extent PIK3CD (z = − 1.1) also reduced the bacterial burden in the MelJuSo-*Mtb* intracellular infection model, while other kinases are potential off-targets^6^. Among the *Mtb* hit compounds, eight structurally related compounds are morpholino-imidazo/triazolo-pyrimidinones and three are 2-aminobenzimidazoles (Fig. 3b). Seven morpholino-imidazo/triazolo-pyrimidinones were found specific for PIK3CB, PIK3CD and VPS34 (Figs. 3c and d). For GSK2373723A, kinase inhibition data were not available, but this compound has also been reported in literature as a PIK3CB and PIK3CD inhibitor^22^. Knockdown of PIK3CB, PIK3CD and VPS34 shows that PIK3CB is the most critical for *Mtb* survival in the MelJuSo cell line (z = − 2.3), while VPS34 activity is dispensable (z = 0.3; Fig. 3e).

The 2-aminobenzimidazoles were originally developed to target VEGFR2 and TIE2 to prevent angiogenesis^23^, but the compounds inhibit 51 kinases by > 50% at 1 µM concentration (Figs. 3f–g, Supplementary Fig. S4). Knockdown of ABL1, AXL, MET, EPHA4 and ZAK reduced the intracellular *Mtb* burden, with ABL1 showing the largest effect (z = − 2.2). Similar to the 2-aminobenzimidazoles, compound GW560109X is a strong inhibitor of ABL1, and its target BLK (z = − 2.1) strongly contributes to its inhibitory effect on intracellular *Mtb* as well (Supplementary Figs. S5a and S6b).

Most remaining *Mtb* hit compounds show a relatively ubiquitous kinase inhibition profiles. One exception is 4-anilinoquinazoline GW576924A, an inhibitor of EGFR, HER2 and HER4 (Supplementary Fig. S5c), which shares its chemotype with previously identified HDTs against intracellular *Mtb,* such as the EGFR inhibitor gefitinib^7–10^. Of these targets, knockdown of HER4 contributes to decreased intracellular *Mtb* burden (z = − 1.1; Supplementary Fig. S5d). Furthermore, compound GSK1379722A mainly inhibits ANKRD3, CSNK1G2, ERK1, GRK3, ICK, IGF1R, and PIK4CB of which knockdown of ANKRD3 (z = − 1.3), IGF1R (z = − 1.7) and PI4KB (z = − 1.3) contribute to reduced *Mtb* burden (Supplementary Figs. S5e and S5f).

In summary, we identified PIK3CB and ABL1 as key host kinases important for the host–pathogen interactions of *Mtb*-infected cells. Additionally, inhibition of AXL, MET, EPHA4, ZAK and BLK, HER4, IGF1R and ANKRD3 by PKIS compounds likely contributed to a reduced bacterial burden as well.

### All *Stm* hit compounds and the majority of *Mtb* hit compounds reduce bacterial counts, acting in a host-directed manner

**Figure 4:**
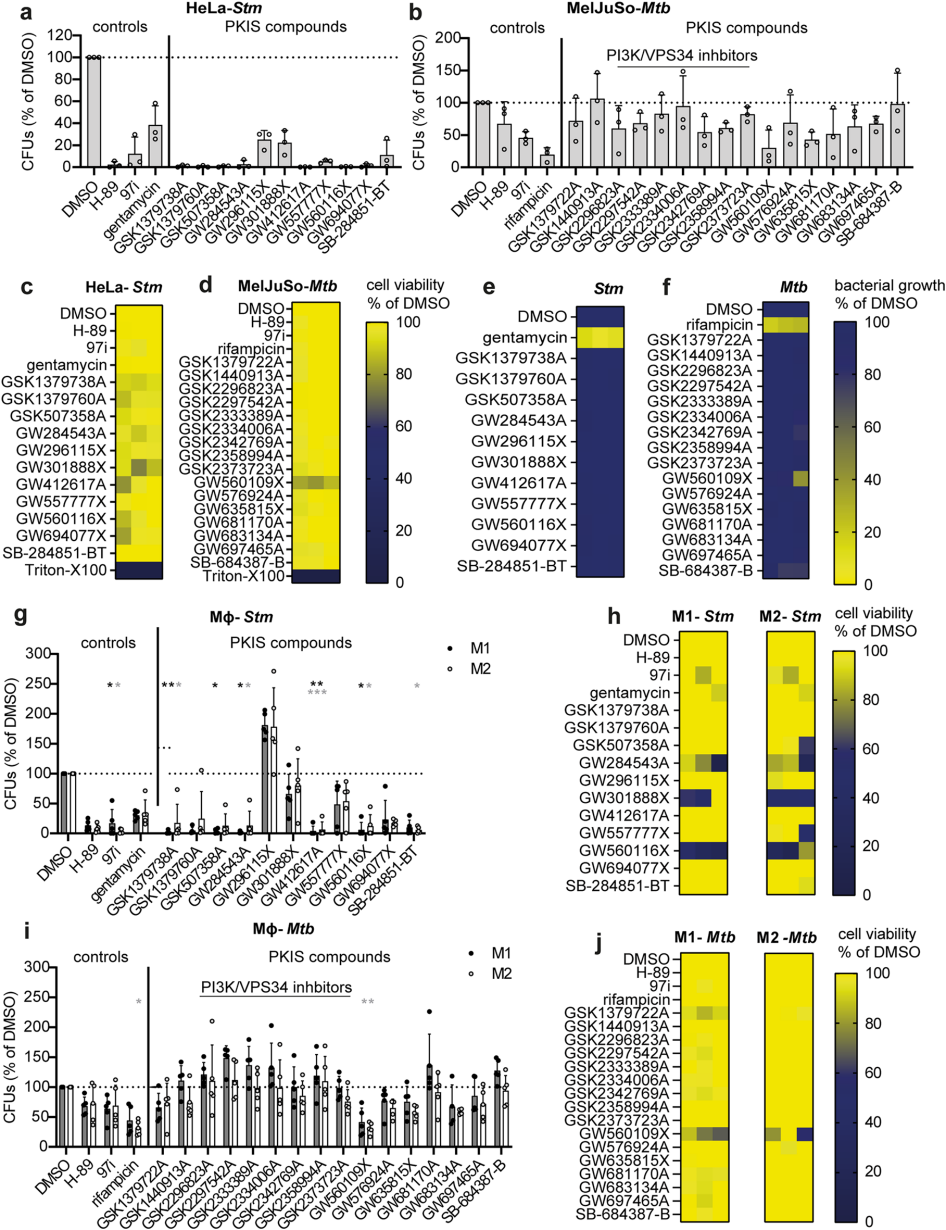
Validation of PKIS hit compounds in cell lines and primary human macrophages. (**a**, **b**) The efficacy of the hit compounds was validated in CFU assays using lysates from *Stm*-infected HeLa cells (**a**) and *Mtb*-infected MelJuSo cells (**b**). The bacterial burden is expressed as a percentage of CFUs compared to the DMSO control. (**c**, **d**) Compound safety was assessed using an LDH-release assay with supernatant from *Stm*-infected HeLa cells (**c**) and *Mtb*-infected MelJuSo cells (**d**), with cell viability expressed as a percentage of the DMSO control and with 1% Triton X-100-treated cells corresponding to 0%. (**e**, **f**) To assess whether hit compounds act as antibiotics or host-directed therapeutics, direct antimicrobial effects were evaluated in cell-free cultures of *Stm* (**e**) and *Mtb* (**f**). The turbidity of the bacterial suspensions, as measured by absorbance at OD_600_, is given as a percentage of the DMSO control. (**g**) The efficacy of the hit compounds was validated in CFU assays using lysates from *Stm*-infected M1 (black circles, grey bars) and M2 (white circles, open bars) primary human macrophages. (**h**) An LDH-release assay was performed using supernatant from *Stm*-infected macrophages. (**i**) The efficacy of the hit compounds was validated in CFU assays using lysates from *Mtb*-infected M1 (black circles) and M2 (white circles) primary human macrophages. (**j**) An LDH-release assay was performed using supernatant from *Mtb*-infected macrophages.

Validation of the efficacy of hit compounds in CFU assays showed that all *Stm* hit compounds effectively reduced the intracellular bacterial burden of *Stm*-infected HeLa cells, with 8 out of 11 compounds that showed > 90% inhibition (Fig. 4a). The *Mtb* hit compounds showed a more modest reduction in intracellular bacterial load with 11 compounds reducing *Mtb* bacterial load > 25% and 2 compounds (*i.e.*, GW560109X and GW635815X) leading to a reduction greater than the 97i positive control (Fig. 4b). The safety of the compounds was validated further by an lactate dehydrogenease (LDH)-release cytotoxicity assay. *Stm* compound GW301888X (Fig. 4c) and *Mtb* compound GW560109X (Fig. 4d) reduced the viability of HeLa and MelJuSo cells by 15 and 17%, respectively, while no noteworthy loss in viability was observed for the other compounds. Finally, we assessed whether the selected *Stm* and *Mtb* hit compounds exerted any direct antibacterial activity on planktonic bacteria to confirm that the compounds act as HDTs. For *Stm*, none of the compounds showed direct effects in planktonic *Stm* culture (Fig. 4e). For *Mtb*, compounds GW560109X and SB-684387-B exhibited some direct effect, reducing the bacterial concentration in planktonic cultures by 27% and 18%, respectively, after 13 days of incubation (Fig. 4f).

### Many identified hit compounds are also effective against *Stm* or *Mtb* infected primary human macrophages, while phosphatidylinositol 3-kinase inhibitors are only effective against *Mtb*-infected MelJuSo cells

Macrophages play a critical role during *Stm* and *Mtb* infections, both as part of the immune response and as the preferred host cell type for the bacteria. Therefore, hit compounds were tested in *Stm*-infected and *Mtb*-infected primary human macrophages. CD14^+^ monocytes were isolated from PBMCs of healthy human donors and the cells were cultured in the presence of GM-CSF and M-CSF to generate M1 and M2 macrophages, respectively (Supplementary Figs. S6a–c). The two types of macrophages were clearly distinguishable by the expression of CD11b, CD163 and CD14, in accordance with previous publications (Supplementary Figs. S6d and S6e)^24^. The majority of the compounds that were active in *Stm*-infected HeLa cells were also active in both types of *Stm*-infected macrophages (Fig. 4g). However, compounds GW296115X, GW301888X and GW557777X showed reduced activity in *Stm*-infected macrophages. In contrast to HeLa cells, in which none of the compounds were found to be cytotoxic, compounds GW284543A (M1: 58.4%; M2: 64.3%), GW301888X (M1: 68.8%; M2: 47.9%) and GW560116X (M1: 34.4%; M2: 41.3%) reduced cell viability of primary human macrophages as compared to the dimethyl sulfoxide (DMSO) solvent control (Fig. 4h). Testing the *Mtb* hit compounds on *Mtb*-infected macrophages showed that many compounds were as active as in *Mtb*-infected MelJuSo cells, with compound GW560109X being the most active in both cell types (Fig. 4i). In addition, the moderate cytotoxicity of compound GW560109X observed in MelJuSo cells was also seen in primary human macrophages (Fig. 4j). In contrast, the group of morpholino-imidazo/triazolo-pyrimidinones acting on PI3K/VPS34 and compound GW681170A were active in *Mtb*-infected MelJuSo cells, but not in primary human macrophages (Figs. 4b and i).

### *Stm* hit compounds readily inhibit intracellular bacterial growth and several display high selectivity indexes in vitro

**Figure 5:**
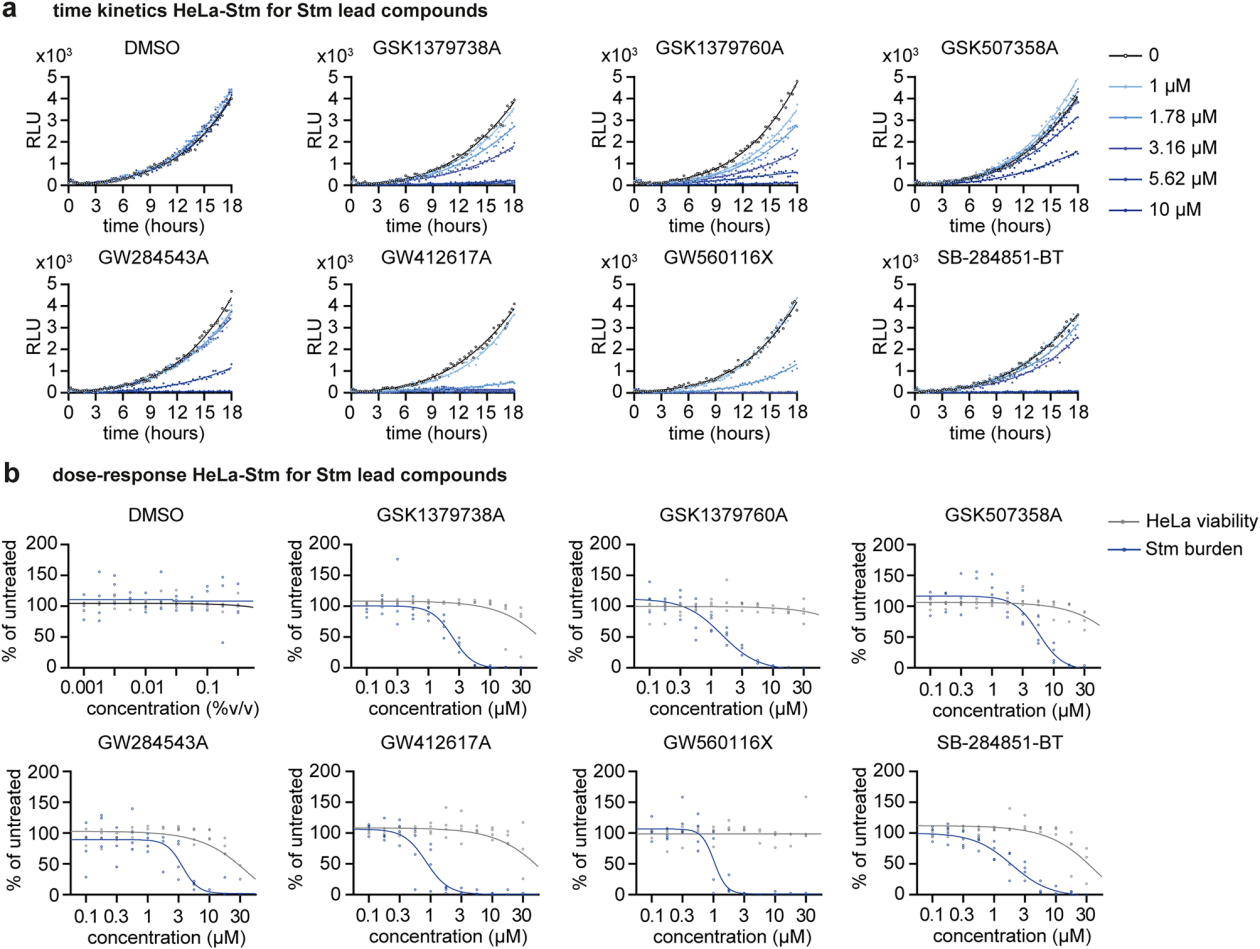
Time kinetics and dose–response relationship of *Stm* hit compounds in *Stm*-infected HeLa cells. (**a**) HeLa cells were infected with bioluminescent *Stm*-lux, treated with *Stm* hit compounds or DMSO at different concentrations and followed over time by measuring emitted light, expressed as relative light units (RLU). DMSO was used at equal % (v/v). Gompertz growth curves were fitted using non-linear regression. (**b**) Bioluminescence, shown in blue, was measured for HeLa cells infected with *Stm*-lux after 18 h of treatment with *Stm* hit compounds at indicated concentrations. RLUs were normalized to the untreated control. Four-parameter logistic regression was used to determine the dose–response relationship between compounds and inhibition of *Stm* growth, and to determine the IC_50_ values of compounds. In addition, the cell supernatant was used to determine host cell viability, shown in grey, using LDH-release assays. Again, four-parameter logistic regression was performed to determine the LD_50_ values of compounds. The data comprises four independent technical replicates.

After validating the effectiveness of many *Stm* hit compounds in infected primary human macrophages, we performed dose-titration experiments on *Stm*-infected HeLa cells for the seven most efficacious compounds to determine their selectivity index, which is important for their potential for further progression. To monitor bacterial growth over 18 h, we performed dose-titration experiments using a bioluminescent *Stm*-lux strain (Fig. 5a). After a short lag phase, during which the bacteria were controlled by the host cells, *Stm* started to grow exponentially. At their effective concentration, all compounds readily inhibited the bacterial growth rate. The minimal inhibitory concentration (MIC) values of the compounds ranged from 1.8 to 14 µM (Table 1). Dose–response curves were created to determine the in vitro potency and safety of the compounds (Fig. 5b). The half maximal inhibitory concentration (IC_50_) values ranged from 1.0 to 6.5 µM and selectivity indexes from 6.5 to 107 (Table 1). Some compounds showed some cytotoxicity at the tested concentrations, with GW284543A being the most cytotoxic, with a half maximal lethal dose (LD_50_) value of 24 µM. Interestingly, compound GW560116X showed no cytotoxicity in HeLa cells, in contrast to primary human macrophages for which significant cytotoxicity was observed at 10 µM concentration (Fig. 4h).

**Table 1:** In vitro efficacy (E_max_), inhibitory concentration required to kill 90% of bacteria (IC_90_), safety (LD_50_) and selectivity index (SI; ratio of IC_90_ to LD_50_) of *Stm* hit compounds against intracellular *Stm*.

Compound	E_max_ (% inh.)	IC_90_ (μM)	LD_50_ (µM)	SI
GSK1379738A	100 (92–100)	5.5 (3.9–9.2)	450 (34–116)	8.2
GSK1379760A	100 (93–100)	5.8 (3.8–9.9)	161 (ND)	28
GSK507358A	100 (87–100)	14.3 (9.7-ND)	490 (ND-156)	3.4
GW284543A	098 (85–100)	7.2 (4.5-ND)	240 (21–27)	3.3
GW412617A	099 (91–100)	2.2 (1.4–4.7)	330 (28–50)	15
GW560116X	099 (90–100)	1.8 (1.3–3.2)	ND	ND
SB-284851-BT	100 (93–100)	7.6 (5.3–12.5)	260 (22–32)	3.4

Numbers between brackets show the 95% confidence interval. Some values could not be determined (ND).

### Structurally related *Stm* hit compounds GSK1379738A and GSK1379760A exhibited significant efficacy in infected zebrafish embryos

**Figure 6:**
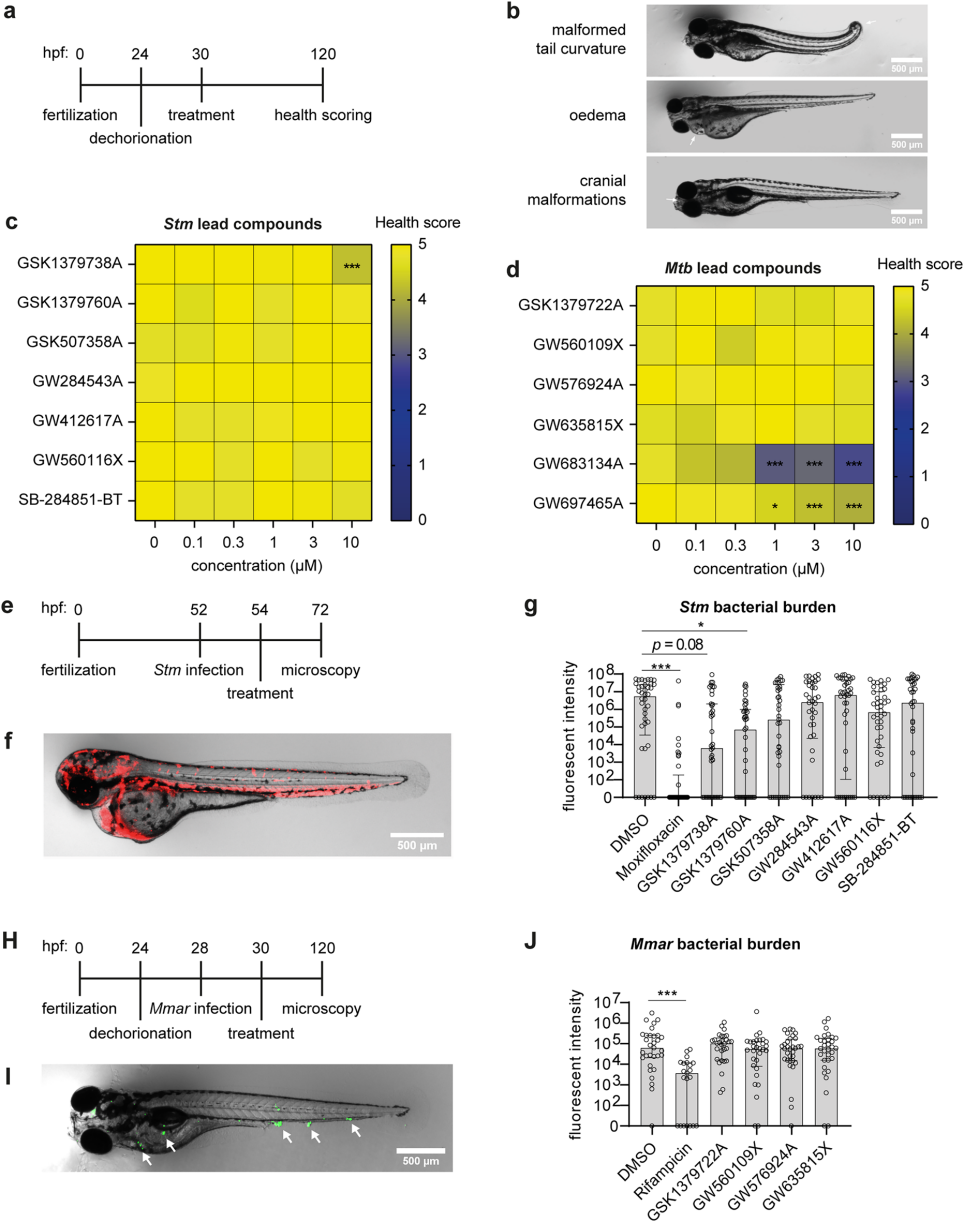
Testing of *Stm* and *Mtb* hit compounds in vivo in zebrafish embryo models. (**a**) Schematic representation of the zebrafish embryo toxicity model. Embryos were visibly inspected and scored for health with a score of 5 representing healthy embryos, 0 representing dead embryos, and scores in between representing embryos with one to four of the following conditions: malformed tail curvature, oedema, cranial malformations, no response to physical stimulation. (**b**) Representative images of embryos with malformations. (**c-d**) Zebrafish embryos were treated with *Stm* (**c**) and *Mtb* (**d**) hit compounds at different concentrations. Each square of the heat map represents the average health score of 20 embryos. (**e**) Schematic representation of the zebrafish embryo *Stm*-infection model. Embryos were infected with ~ 200 CFUs at 52 h post fertilization (hpf). (**f**) Representative image of *Stm*-infected zebrafish embryos treated with DMSO at 72 hpf. (**g**) *Stm*-infected zebrafish were treated with 0.1% (v/v) DMSO, 1 µM moxifloxacin, 3 µM GSK1379738A and other compounds at 10 µM. Each group comprises 38–42 embryos. (**h**) Schematic representation of the zebrafish embryo *Mmar*-infection model. Embryos were infected with ~ 200 CFUs at 28 hpf. (**I**) Representative image of *Mmar*-infected zebrafish embryos treated with DMSO at 120 hpf. (**J**) *Mmar*-infected embryos were treated with 0.1% DMSO, 200 µM rifampicin and other compounds at 10 µM. Each group comprises 21–32 embryos. Statistically significant differences are indicated by *p < 0.05 and ***p < 0.001.

Finally, we determined whether the seven most effective *Stm* compounds and the six most effective *Mtb* compounds were safe and efficacious in vivo using zebrafish embryo models. In vivo toxicity was determined by administering the compounds directly to the water of dechorionated zebrafish embryos (Fig. 6a). After 4 days the health of the embryos was scored based on their mortality rate and drug-induced side effects (Fig. 6b). For the *Stm* compounds, only GSK1379738A resulted in some toxicity, causing oedema (4/20) at 10 µM concentration (Fig. 6c). *Mtb* compound GW683134A caused oedema and cranial malformations in the majority of embryos starting at 0.3 µM and 1 µM, respectively (Fig. 6d). In addition, GW697465A caused oedema in a large proportion of the embryos starting at 1 µM. The other *Mtb* compounds were considered non-toxic in vivo. Overall, mortality was low even for the toxic compounds at the tested concentrations.

The efficacy of the compounds was tested in zebrafish embryo infection models. Zebrafish embryos were injected with 200 CFUs *Stm*, treated with the *Stm* compounds, and the bacterial burden was quantified 20 h later (Figs. 6e and f). Of note, compound GSK1379738A was used at 3 µM instead of 10 µM, as used for the other compounds, to prevent toxicity. Compounds GSK1379738A, GSK1379760A and GSK507358A showed efficacy, resulting in 870-fold, 77-fold and 21-fold reductions in median bacterial burden, respectively, as compared to DMSO-treated controls (Fig. 6g). Due to variation between individuals, only GSK1379760A led to a statistically significant reduction. Next, zebrafish embryos were injected with approximately 200 CFUs *Mycobacterium marinum* (*Mmar*), an established model of human *Mtb* infections^25,26^, to test the in vivo efficacy of the non-toxic *Mtb* compounds GSK1379722A, GW560109X, GW576924A and GW635815X upon 4 days of treatment (Fig. 6h). Granuloma formation was clearly observed in infected embryos (Fig. 6i). Rifampicin was used as a positive control and resulted in a 29-fold reduction in median bacterial burden compared to the DMSO control (Fig. 6j). However, none of the *Mtb* compounds significantly reduced the *Mmar* bacterial burden. Taken together, most PKIS compounds with activity against intracellular *Stm* and *Mtb *in vitro were found safe in vivo. Furthermore, two structurally related 2-anilino-4-pyrrolidinopyrimidines, GSK1379738A and GSK1379760A, reduced the *Stm* bacterial burden in vivo, showing the potential of this chemotype to use as HDT during *Stm* infections.

## Discussion

In response to the rise in antimicrobial resistance, we aimed to identify new HDTs that may be used as adjunctive or alternative treatments to classical antibiotics. Starting with 827 ATP-competitive kinase inhibitors from the PKIS library, we performed two consecutive screens in intracellular HeLa-*Stm* and MelJuSo-*Mtb* infection models. The bacteria used in the screen expressed DsRed, a fluorescent protein that can well withstand low pH, resulting in effective detection of bacteria within endolysosomal intracellular compartments^27^. Consequently, this screening method primarily detects bacterial growth inhibition, which may or may not involve bacterial degradation. The screen resulted in 11 *Stm* hit compounds and 17 *Mtb* hit compounds that were further validated. All active compounds were found non-cytotoxic and to act in a host-directed manner. In line with previous findings, there was no overlap between *Stm* and *Mtb* hit compounds, or their kinase targets, suggesting that these intracellular pathogens likely depend on very different signaling pathways to persist intracellularly^6^. Importantly, most *Stm* and multiple *Mtb* hit compounds were also active in primary human macrophages, which play a key role in the pathogenesis of these bacteria, both as host cells and effector immune cells^28,29^. Moreover, we were able to demonstrate that two related *Stm* compounds, GSK1379738A and GSK1379760A, effectively reduced the *Stm* bacterial burden in infected zebrafish embryos.

The PKIS hit compounds against *Mtb* largely comprised a group of morpholino-imidazo/triazolo-pyrimidinones targeting PIK3CB, PIK3CD and VPS34. Of these, PIK3CB and to a lesser extent PIK3CD were found to affect intracellular *Mtb* persistence in MelJuSo cells upon knockdown^6^. The higher efficacy of the morpholino-imidazo/triazolo-pyrimidinones in MelJuSo cells as compared to primary human macrophages may be explained by the presence of a gain-of-function mutation in the NRAS gene at c.182A > T in MelJuSo cells, resulting in constitutively increased activity of PI3K^30^. PIK3CB and PIK3CD phosphorylate phosphatidylinositol 4,5-bisphosphate to create phosphatidylinositol 3,4,5-trisphosphate at the plasma membrane, which provides a binding site for many cytosolic proteins including AKT. Previous studies have demonstrated the involvement of the PI3K/AKT pathway for intracellular persistence of both *Stm* and *Mtb*^6,8,14,31,32^. In this study, PI3K activity clearly facilitated persistence of *Mtb* in infected MelJuSo cells, but seemingly had no effect in *Mtb*-infected primary human macrophages or *Stm*-infected HeLa cells. Vice versa, inhibition of AKT by H-89 and GSK507358A had limited activity on *Mtb*-infected MelJuSo cells, but potently inhibited *Stm* in infected HeLa cells or primary human macrophages. Thus our results suggest that while PI3K/AKT kinases are important in both *Stm* and *Mtb* infection, the exact kinases involved in bacterial persistence are specific to both pathogen and host cell.

In addition, we identified 2-aminobenzimidazoles targeting ABL1 as *Mtb* hit compounds, in line with previous studies that demonstrated that targeting ABL1 by HDTs improved lysosome maturation and impaired *Mtb* replication in infected cells^6,11–15,33^. Of note, an inhibitor of ABL1, imatinib, is currently tested in clinical trials as adjunctive therapy together with an antibiotic regimen of rifabutin and isoniazid against drug-sensitive TB^16^. In addition, compound GW560109X, a structurally unrelated inhibitor of ABL1, as well as BLK, was active against intracellular *Mtb*, both in infected MelJuSo cells and primary human macrophages^18^. Other interesting compounds that were identified against intracellular *Mtb* included the EGFR kinase family inhibitor GW576924A and IGF1R inhibitor GSK1379722A, which is consistent with other studies showing that inhibition of these kinase targets stimulates autophagy and lysosome biogenesis, resulting in killing of intracellular *Mtb*^7–10,33^.

The *Stm* hit compounds were part of diverse chemotypes with only a few targets in common. Compounds GSK1379738A and GSK1379760A are both 2-anillino-4-pyrrolidinopyrimidines that target AAK1, involved in clathrin-mediated endocytosis, and JAK2, involved in JAK/STAT signal transduction downstream of cytokine receptor activation. Previous studies have shown that JAK-independent activation of STAT3 by the secreted *Stm* SarA virulence factor stimulates the formation of *Salmonella*-induced filaments and *Stm* replication^34,35^. Furthermore, pimozide was identified as a host-directed therapeutic against intracellular *Stm* acting on STAT5^36^. Our results indicate that inhibition of JAK2 by GSK1379738A and GSK1379760A also inhibits replication of intracellular *Stm* in infected HeLa cells and primary macrophages. The 4-anilinoquinoline *Stm* hit compounds shared many kinase targets, several of which were found to be involved in *Stm* intracellular persistence, including MAP2K5, RIPK2 and RSK4. Inhibition of RIPK2 is known to dampen the inflammatory response against invading *Stm*^37^. GSK507358A probably acts by stimulating *Stm* trafficking to the lysosome through inhibition of AKT, as previously described for H-89^14^.

Seven *Stm* hit compounds and six *Mtb* hit compounds were tested in vivo in zebrafish embryo models, of which compounds GSK1379738A and GSK1379760A clearly reduced *Stm* burden. These compounds are structurally closely related, showing that this chemotype is interesting to further optimize for use as HDT. Possibly, GSK1379738A and GSK1379760A act by targeting JAK2 or AAK1, which are shared kinase targets. In addition, the AKT inhibitor GSK507358A reduced the *Stm* bacterial burden by 20-fold, but this did not reach statistical significance due to the interindividual variation. This study is one of the first to demonstrate that the *Stm* zebrafish infection model can serve as an early in vivo model to identify promising HDTs. Current limitations of this model are that the course of infection occurs very fast and is highly dependent on the starting inoculum. During optimization experiments, too few bacteria in the starting inoculum resulted in early clearance of the bacteria, whereas too many bacteria led to uncontrolled replication extracellularly followed by the early death of the zebrafish embryos. Future optimization of this model may include the use of suboptimal doses of antibiotics to create a more stable course of infection that allows a longer period of observance.

While the zebrafish model was successfully employed to confirm the efficacy of *Stm* compounds, none of the *Mtb* compounds showed efficacy in the zebrafish TB model, possibly because the model used Mmar, a closely related but different pathogen. In addition, we exposed the embryos to the drugs by immersing them in compound-supplemented water, a commonly used method, which, however, is not suitable for poorly absorbed drugs^38,39^. This may also explain why the in vitro cytotoxicity data based on LDH-release assay was not found predictive for in vivo toxicity. For example, 10 µM *Stm* hit compound GW560116X and 10 µM *Mtb* hit compound GW560109X were cytotoxic when administered to primary human macrophages in vitro, but did not result in any side effects in zebrafish embryos. In future experiments, the route of administration of HDTs may have to be optimized depending on their absorbance.

In summary, we identified morpholino-imidazo/triazolo-pyrimidinones, targeting PIK3CB and PIK3CD, as interesting compounds against intracellular *Mtb* and found that their effect was cell type-specific, showing reduced activity in primary human macrophages as compared to the MelJuSo cell line. Moreover, we identified 2-aminobenzimidazoles, targeting receptor tyrosine kinase ABL1, as active against intracellular *Mtb*. Interestingly, these kinases were previously identified as targets for HDT against *Mtb*^6^. For *Stm*, we identified 7 compounds that were highly potent in both *Stm*-infected HeLa cells and primary human macrophages, resulting in near clearance of the intracellular bacterial burden. Compound GSK1379760A, a 2-anilino-4-pyrrolidinopyrimidine, was found to be the most potent kinase inhibitor against intracellular *Stm*, showing a selectivity index of > 107-fold in vitro and reducing the bacterial burden by 77-fold in zebrafish embryos in vivo. Another 2-anilino-4-pyrrolidinopyrimidine, compound GSK1379738A, was active against *Stm*, both in vitro and in vivo, which demonstrates the potential of this chemotype for use as HDT. This study furthermore demonstrates the capacity of the exploited pipelines to identify potent HDTs that are translatable and well tolerated in vivo. Further studies are required to demonstrate the potential of GSK1379760A as an adjunctive or alternative treatment for antibiotics, including its ability to treat bacterial infection in vivo in mammals and humans.

## Materials and methods

### Ethical statements

Human blood samples were isolated from buffy coats obtained from healthy donors after written informed consent (Sanquin, Amsterdam, the Netherlands). The biological samples were sourced ethically and their research use was in accordance with the terms of the informed consents under an IRB/EC approved protocol. The use of blood samples was approved by the Sanquin Ethical Advisory Board and in accordance with the Declaration of Helsinki. The husbandry of adult zebrafish lines and the experimental protocols described in this study was in accordance with guidelines from the local animal welfare body (AWB) of Leiden University (License number: protocol 14,198), and in compliance with the international guidelines specified by the EU Animal Protection Directive 2010/63/EU. Experiments with zebrafish embryos were performed within 5 days post fertilization and therefore did not involve any procedures within the meaning of Article 3 of Directive 2010/63/EU. The reported results are in compliance with ARRIVE guidelines.

### Reagents

Kinase inhibitor H-89 dihydrochloride was purchased from Sigma-Aldrich (Merck, Darmstadt, Germany). The H-89 analogue 97i was a gift from prof. dr. M. van der Stelt and prepared *in house* according to a previous publication^21,40^. Rifampicin, moxifloxacin hydrochloride, gentamycin sulfate, DMSO and Triton X-100 were all purchased from Sigma-Aldrich. A total of 827 kinase inhibitors from the published kinase inhibitor sets (PKIS)1^17^ and PKIS2^18^ was obtained from GlaxoSmithKline Global Health Medicines R&D (GSK) and the Structural Genomics Consortium of the University of North Carolina at Chapel Hill (SGC-UNC). All kinase inhibitors, including H-89 and 97i, were dissolved at 10 mM concentration in DMSO. Mouse anti-human antibodies CD14-FITC (clone 63D3), CD163-PE (clone GHI/61), CD14-PE/Cy7 (clone 63D3) and CD1a-AF647 (clone HI149) were purchased from Biolegend (San Diego, CA, USA). Mouse anti-human CD11b-BB515 (clone ICRF44) was purchased from BD Biosciences (Franklin Lakes, NJ, USA).

### Cell culture

HeLa epithelial cells and MelJuSo human melanoma cells were cultured in Gibco Iscove’s Modified Dulbecco’s Medium (IMDM; ThermoFisher Scientific, the Netherlands) supplemented with 10% fetal bovine serum (FBS; Greiner Bio-One, Alphen a/d Rijn, the Netherlands), 100 units/ml Gibco penicillin and 100 µg/ml Gibco streptomycin (both from ThermoFisher Scientific) at 37 °C/5% CO_2_.

Primary human macrophages were generated by differentiating blood-derived CD14^+^ monocytes for 6 days in Roswell Park Memorial Institute (RPMI)-1640 medium (ThermoFisher Scientific) containing 10% HyClone FBS (Cytiva, MA, Marlborough, USA), 100 units/ml penicillin, 100 µg/ml streptomycin and either 5 ng/ml granulocyte–macrophage colony-stimulating factor (GM-CSF; R&D Systems, Abingdon, UK) to promote M1-differentiation or 20 ng/ml macrophage colony-stimulating factor (M-CSF; R&D Systems) to promote M2-differentiation as previously described^6^. Macrophages were collected by incubation in Gibco Trypsin–EDTA solution (ThermoFisher Scientific) and gentle scraping. The M1 and M2 macrophage phenotypes were validated based on morphology and surface marker expression using flow cytometry with M1 macrophages being CD14^low^, CD163^low^ and CD11b^high^, whereas M2 macrophages were CD14^high^, CD163^high^, CD11b^low^^24^.

### Bacterial culture

*Stm* strain SL1344 with plasmid pMW211[C.10E/DsRed.T3_S4T]^41^ and strain 12,023 with plasmid pluxCDABE^14^ were recovered from frozen glycerol stock and cultured in Difco Luria–Bertani (LB) Broth (BD Biosciences) containing 100 µg/ml ampicillin (Merck, Darmstadt, Germany) overnight at 37 °C in a shaking incubator. *Stm* was subcultured 1:33 three to four hours prior to infection to obtain a log-phase bacterial culture. *Mtb* strain H37Rv with plasmid pSMT3[Phsp60/DsRed.T3_S4T]^6^ was cultured at 37 °C in complete Difco Middlebrook 7H9 Broth (BD Biosciences), supplemented with 10% ADC (BD Biosciences), 0.05% Tween-80 (Sigma–Aldrich), 0.2% glycerol (Sigma–Aldrich) and 50 µg/ml Gibco hygromycin (ThermoFisher Scientific) in a shaking incubator. *Mtb* was split once a week to maintain log-phase bacterial cultures. *Mmar* strain M with plasmid pTEC15[mWasabi]^42^ was diluted to an OD_600_ of 0.1 the day before infection and cultured overnight complete 7H9 medium at 28.5 °C in a static incubator.

### *Stm *and *Mtb* intracellular infection and HDT treatment

Intracellular infection experiments were performed as previously described with minor modifications^6,36,43–46^. HeLa or MelJuSo cells were resuspended in IMDM with 10% FBS without antibiotics and seeded with 10,000 cells/well and 9000 cells/well, respectively, into Costar flat-bottom 96-well plates (Corning, Amsterdam, the Netherlands). M1 and M2 macrophages were resuspended in RPMI with 10% FBS without antibiotics and seeded with 30,000 cells/well into Costar flat-bottom 96-well plates. The cells were incubated overnight at 37 °C/5% CO_2_. The next day, cells were inoculated with log-phase bacterial suspensions in cell culture medium at an expected multiplicity of infection (MOI) of 10. Accuracy of the MOI was validated by plating serial dilutions of the *Stm* and *Mtb* inoculums on Difco LB agar plates and Middlebrook 7H10 agar plates supplemented with 10% OADC and 0.5% glycerol, respectively (all from BD Biosciences). The observed MOIs were 11.6 (n = 18; range 3.6–15.9) and 18.9 (n = 13; range 5.2–41.1), respectively. Plates with infected cells were centrifuged for 3 min at 150 *g* and incubated for 20 min for *Stm* infection or 1 h for *Mtb* infection at 37 °C/5% CO_2_. Extracellular bacteria were removed by incubation with fresh cell culture medium supplemented with 30 µg/ml gentamicin sulphate (Lonza BioWhittaker, Basel, Switzerland) for 15 min at 37 °C/5% CO_2_. Infected cells were incubated overnight at 37 °C/5% CO_2_ in cell culture medium in the presence of kinase inhibitors at 10 µM concentration and 5 µg/ml gentamycin sulphate-containing medium to prevent growth of extracellular bacteria. Kinase inhibitors H-89 and its derivative 97i were used at 10 µM concentrations as HDT positive controls for *Stm* and *Mtb*, respectively, and an equal amount of DMSO (% v/v), used as solvent for all compounds, was used as a negative control. Rifampicin and gentamycin were used at 1 µM and 30 µg/ml, respectively.

### Compound screens by flow cytometry

Flow cytometry-based screens were performed in line with a previous publication^6^. In summary, HeLa and MelJuSo cells were harvested by trypsinization and fixed with 1% paraformaldehyde. Samples from the primary screen were measured on a FACSCalibur with high-throughput samples (HTS) extension and samples from the rescreen were measured on a FACSLyric (BD Biosciences) with HTS extension (all from BD Biosciences). Flow cytometry data were analysed using FlowJo version 10 (TreeStar, Ashland, OR, USA). In the primary screens, each plate contained H-89 and DMSO as positive and negative controls, and each plate was tested in triplicate. In the rescreens, we included H-89-derivative 97i as a positive control, since 97i was recently shown to be more active against both intracellular *Mtb* and *Stm* than H-89^21^. *Mtb*-infected MelJuSo cells positive for DsRed were gated to calculate the percentage of infected cells. For *Stm*-infected HeLa cells, a DsRed-bright population could be distinguished from a DsRed-dim population (Supplementary Fig. S1a). Both the total *Stm*-DsRed+ population and the DsRed-bright population were initially used as readouts for the primary screen, but eventually the DsRed-bright population was used for selection of hit compounds since this population was found to contain nearly all intracellular bacteria after FACS sorting (see methods below; Supplementary Fig. S2). The cell count was used as readout of cell viability.

### Data analysis of flow cytometry screens

Standard z-scores were calculated from the flow cytometry data to identify candidate ‘hit’ compounds by z = (x-μ)/σ—z_neg_, where x is total event count or the percent of DsRed-bright cells or DsRed+ cells from a single well, μ is the mean from wells of the plate, σ is the standard deviation of the plate and z_neg_ is the mean z-score of the negative control of the plate (i.e. DMSO). The subtraction of z_neg_ was used to correct z-scores for plate-to-plate variations in baseline infections rates or cell viability. Values that deviated two-fold from the plate mean were excluded to calculate the plate mean and standard deviation as used in the aforementioned formula, to minimize the possibility that extreme values affected the plate mean and standard deviation. Compounds that had a Z-score < − 3 for cell count were considered cytotoxic and were therefore excluded as hit compounds. Compounds with a z-score below or above the critical value of respectively − 2 or 2 for DsRed+ and/or DsRed-bright events and a z-score above the critical value of − 3 for cell count were considered ‘hit’ compounds. However, after the rescreen only compounds with a DsRed z-score below the critical value of − 2 were further analysed.

### Identification of kinase targets

Kinase inhibition data at 1 µM concentration have previously been determined for the PKIS compounds, except for GSK2373723A, in biochemical assays by others^17,18^. KinMap phylogenetic trees of protein kinase families were generated at https://kinhub.org/kinmap/ for visual representation of kinase inhibition data^47^. A phylogenetic tree of phosphatidylinositol kinases was created by multiple sequence alignment using Clustal Omega at https://www.ebi.ac.uk/Tools/msa/clustalo/, based on the PROSITE-predicted kinase catalytic domains, and neighbor-joining at https://icytree.org/^48^. Most PKIS compounds target multiple kinases. The contribution of individual kinases to host–pathogen interactions was determined by a dataset from previously reported kinome siRNA knockdown screens in which the same HeLa-*Stm* and MelJuSo*-Mtb* intracellular infection models have been used^6^.

### Colony-forming unit assay

Infected cells were lysed in sterile water + 0.05% Invitrogen UltraPure SDS solution (ThermoFisher Scientific) to release bacteria. Bacterial suspensions and lysates of cells infected with *Stm* and *Mtb* were fivefold serially diluted and 10-μl drops were plated on square agar plates, using Difco LB agar plates for *Stm* and Middlebrook 7H10 agar plates for *Mtb*. The plates were left to dry and incubated overnight at 37 °C/5% CO_2_ for *Stm* and approximately two weeks for *Mtb*, after which CFUs were counted manually.

### FACS sorting

*Stm*-infected HeLa cells were FACS-sorted in DsRed-dim and DsRed populations using the CytoFLEX SRT II cell sorter (Beckman Coulter Fullerton, CA, USA) of the Flow Cytometry Core Facility of the Leiden University Medical Centre to determine the intracellular bacterial burden of both populations. After sorting, the cells were lysed, and the lysate was plated out on LB agar plates to determine the CFU count, as described above.

### Bacterial growth assay

*Stm* or *Mtb* cultures at a concentration corresponding to an absorbance of 0.1 at 600 nm wavelength were incubated with the kinase inhibitors at 10 μM in flat-bottom 96-well plates. The absorbance was measured using an EnVision plate reader (PerkinElmer, Waltham, MA, USA). The plates were incubated at 37 °C overnight for *Stm* and for a period of 15 days for *Mtb*. The absorbance was measured again the next day for *Stm* and every two days for *Mtb*.

### Lactate dehydrogenase release cytotoxicity assay

Before harvesting or lysing cells of intracellular infection assays, supernatant from the cells was collected and used to quantify LDH release using the LDH cytotoxicity detection kit (Roche, Merck, Darmstadt, Germany) according to the manufacturer’s instructions. Quantification was performed with an EnVision plate reader or SpectraMax i3x (Molecular Devices, San Jose, CA, USA) plate reader by using the absorbance at 485 nm as signal and the absorbance at 690 nm as reference wavelength for background subtraction. The DMSO solvent control was used as negative control. Triton X-100 results in maximum LDH release by permeabilizing the cells and was used as positive control. The cell viability was calculated using the following formula: $\left(1-\frac{{A}_{sample}-{A}_{DMSO}}{{A}_{Triton X-100}-{A}_{DMSO}}\right)*100\%$ with *A* for absorbance at 485 nm after subtraction of the absorbance at 690 nm.

### Bioluminescent bacterial growth assay

Upon infection of HeLa cells with *Stm*-lux, bacterial growth was followed over time by incubating the cells in the SpectraMax i3x plate reader at 37 °C and measuring the bioluminescence every 15 min for 18 h.

### Zebrafish husbandry

For this study, outbred AB/TL zebrafish of both sexes were used. Zebrafish were handled in compliance with animal welfare regulations and maintained according to standard protocols (http://zfin.org). Fertilized embryos were maintained at 28 °C and kept in egg water containing 60 μg/ml Instant Ocean Sea Salt (Sera, Heinsberg Germany). Zebrafish larvae were anesthetized with egg water containing 0.02% buffered ethyl 3-aminobenzoic acid ethyl ester (Tricaine, Sigma-Aldrich, Netherlands) for bacterial infection and imaging experiments.

### Zebrafish embryo toxicity test

Zebrafish embryos were manually dechorionated at around 24 h post fertilization (hpf) to exclude any protective effects from the egg shells. At 28 hpf, the compounds, dissolved in DMSO at 10 mM, were added to the egg water at concentrations ranging between 0.1 and 10 µM. At 120 hpf, the embryos were visually inspected using a Leica M205 FA stereo fluorescence microscope. The health of the embryos was scored with a maximum score of 5 (*i.e.*, healthy). Scores were reduced by 1 point redrawn for the absence of a response to mechanical stimulation, oedema, tail curvature malformations, or cranial malformations. Dead embryos without a heartbeat were given a score of 0.

### Zebrafish embryo infection models

To test *Stm* compounds for efficacy in vivo, embryos that had already hatched from the egg at 48 hpf were collected and systemically infected with *Stm*-DsRed at 52 hpf by injecting 200 CFUs in the Duct of Cuvier as previously described^49^. Infected zebrafish embryos were treated with compounds at 54 hpf. The bacterial burden was quantified at 72 hpf by measuring fluorescence using the Leica M205 FA microscope. To test *Mtb* compounds for efficacy in vivo, dechorionated zebrafish embryos were systemically infected with *Mmar*-mWasabi at 28 hpf by injecting approximately 200 CFUs in the blood island (*i.e.*, caudal vein area), as previously described^49^. The compounds were added to the egg water at 30 hpf. The bacterial burden was determined at 144 hpf by fluorescence using the Leica M205 FA microscope and quantified using Fiji software^50^.

### Statistical analyses

Statistical testing was performed using GraphPad Prism 9 (GraphPad Software, San Diego, CA, USA). Differences in kinase inhibition between hit compounds and non-hit compounds were tested for statistically significant differences using Mann–Whitney tests. The results of intracellular infection and bacterial growth assays were tested for statistically significant differences between treatment and DMSO groups by performing Friedman tests for matched samples and Dunn’s multiple comparisons tests for *post-hoc* analysis. The results of zebrafish experiments were tested for statistically significant differences between treatment and control groups (either DMSO or untreated) by performing Kruskal–Wallis tests for independent samples and Dunn’s multiple comparisons tests for *post-hoc* analysis. Differences between groups resulting in *p* values < 0.05 were considered statistically significant.

### Supplementary Information

Supplementary Information 1.

Supplementary Information 2.

Supplementary Information 3.

## Data Availability

The original contributions presented in the study are included in the article/Supplementary Material. Further inquiries can be directed to the corresponding authors.
