# Supplementary material for: Identification of kinase inhibitors as potential host-directed therapies for intracellular bacteria
Source: Sci Rep. 2024 Jul 26;14:17225. doi: 10.1038/s41598-024-68102-6 (PMC11282061; doi:10.1038/s41598-024-68102-6)
Supplement: Supplementary file 1 — Supplementary Information 1. [file 41598_2024_68102_MOESM1_ESM.pdf]

Identification of kinase inhibitors as potential host-directed therapies for intracellular bacteria

Running title: Kinase inhibitors as HDT for intracellular bacteria

Robin H.G.A. van den Biggelaar[1,2*], Kimberley V. Walburg[1], Susan J.F. van den Eeden[1], Cassandra L.R. van Doorn[1], Eugenia Meiler[3], Alex S. de Ries[2], M. Chiara Fusco[2], Annemarie H. Meijer[2], Tom H.M. Ottenhoff[1], Anno Saris[1*]

[1]Leiden University Center for Infectious Diseases, Leiden University Medical Center, Leiden, the Netherlands

[2]Institute of Biology Leiden, Leiden University, Leiden, the Netherlands

[3]Global Health Medicines R&D, GlaxoSmithKline, Tres Cantos, Spain

***Correspondence:**

Dr. Robin H.G.A. van den Biggelaar, r.h.g.a.van_den_biggelaar@lumc.nl

Dr. Anno Saris, a.saris@lumc.nl

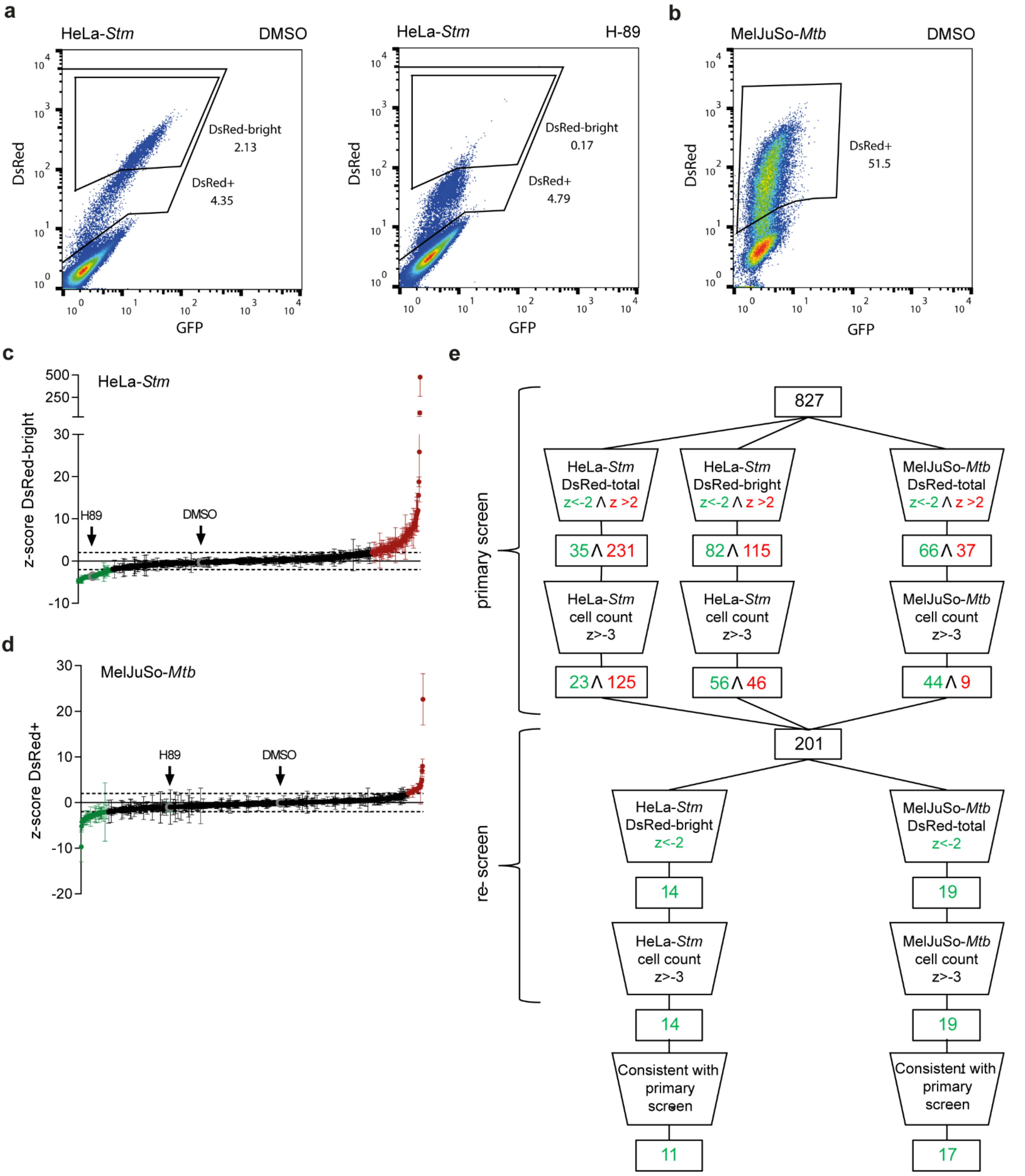

Fig. S1 Screening strategy and primary screen of PKIS compounds affecting *Stm* and *Mtb* intracellular burden. (**a**) Gating strategy for DsRed+ and DsRed-bright *Stm*-infected HeLa cells. H-89-treated cells illustrate that the DsRed-bright population may be reduced without reducing the DsRed+ population. (**b**) Gating strategy for DsRed+ *Mtb*-infected MelJuSo cells. (**c**) Primary screen of 827 PKIS compounds to assess their impact on *Stm* bacterial burden, expressed both as average z-scores of the DsRed-bright population. Compounds with z-scores < -2 or > 2 are shown in green and red, respectively. (**d**) Similar to (c), with z-scores representing intracellular *Mtb* burden. (**e**) Flowchart displaying the strategy that was used to identify non-cytotoxic hit compounds with activity against intracellular *Stm* and *Mtb* from the PKIS compound library.

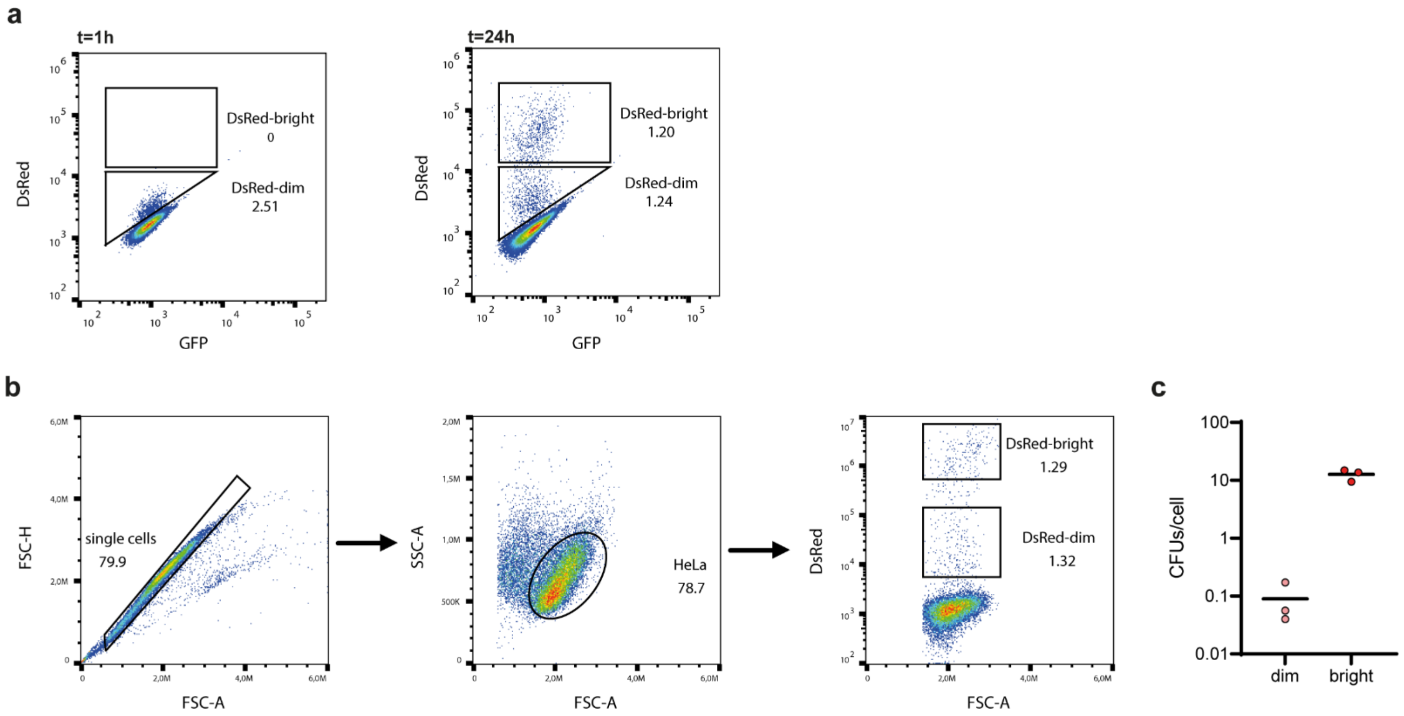

Fig. S2 Comparison DsRed-dim and DsRed-bright *Stm*-infected HeLa cells. (**a**) HeLa cells were fixed either 1 h or 24 h after infection with *Stm*-DsRed to determine differences in the presence of both DsRed populations. (**b**) HeLa cells were selected for FACS sorting based on gates for single cells, size and DsRed fluorescence. (**c**) The DsRed-dim and DsRed-bright populations were sorted and lysed to determine the intracellular bacterial burden by CFU count.

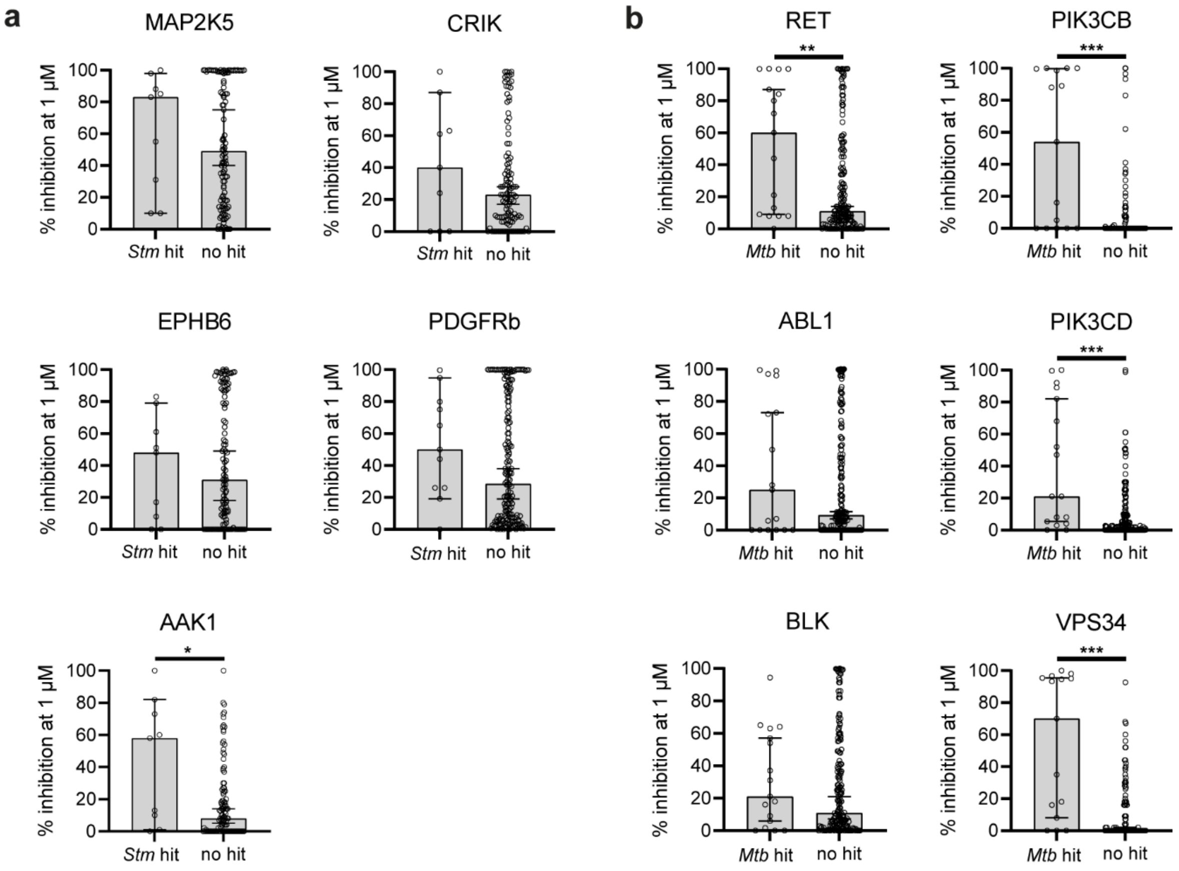

Fig. S3 Comparison of kinase inhibition data between PKIS compounds that reduced the intracellular burden and compounds that did not. (**a**) The level of inhibition of the five most targeted kinases by *Stm* hit compounds was compared with other compounds. (**b**) The level of inhibition of the six most targeted kinases by *Mtb* hit compounds was compared with other compounds. Statistically significant differences are indicated by *$p < 0.05$, **$p < 0.01$ and ***$p < 0.001$.

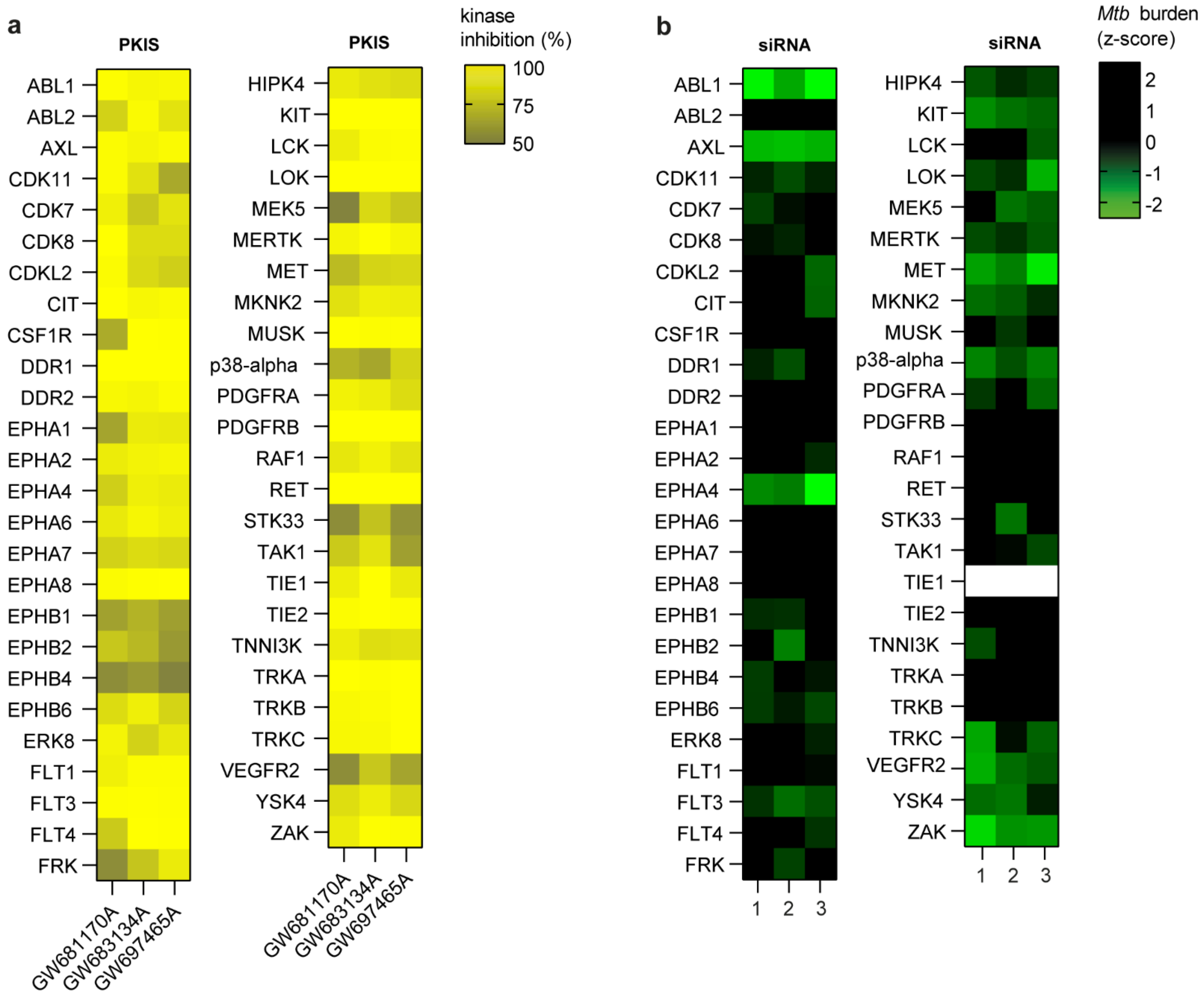

Fig. S4 Full kinase target profile of *Mtb* hit compounds belonging to of 2- aminobenzimidazole chemotype. (**a**) Inhibition of all shared kinase targets of 2-aminobenzimidazole *Mtb* hit compounds. (**b**) Effect of siRNA knockdown of the kinase targets depicted in (a) on the bacterial burden of *Mtb*-infected MelJuSo cells. White squares represents targets for which genetic inhibition was not available.

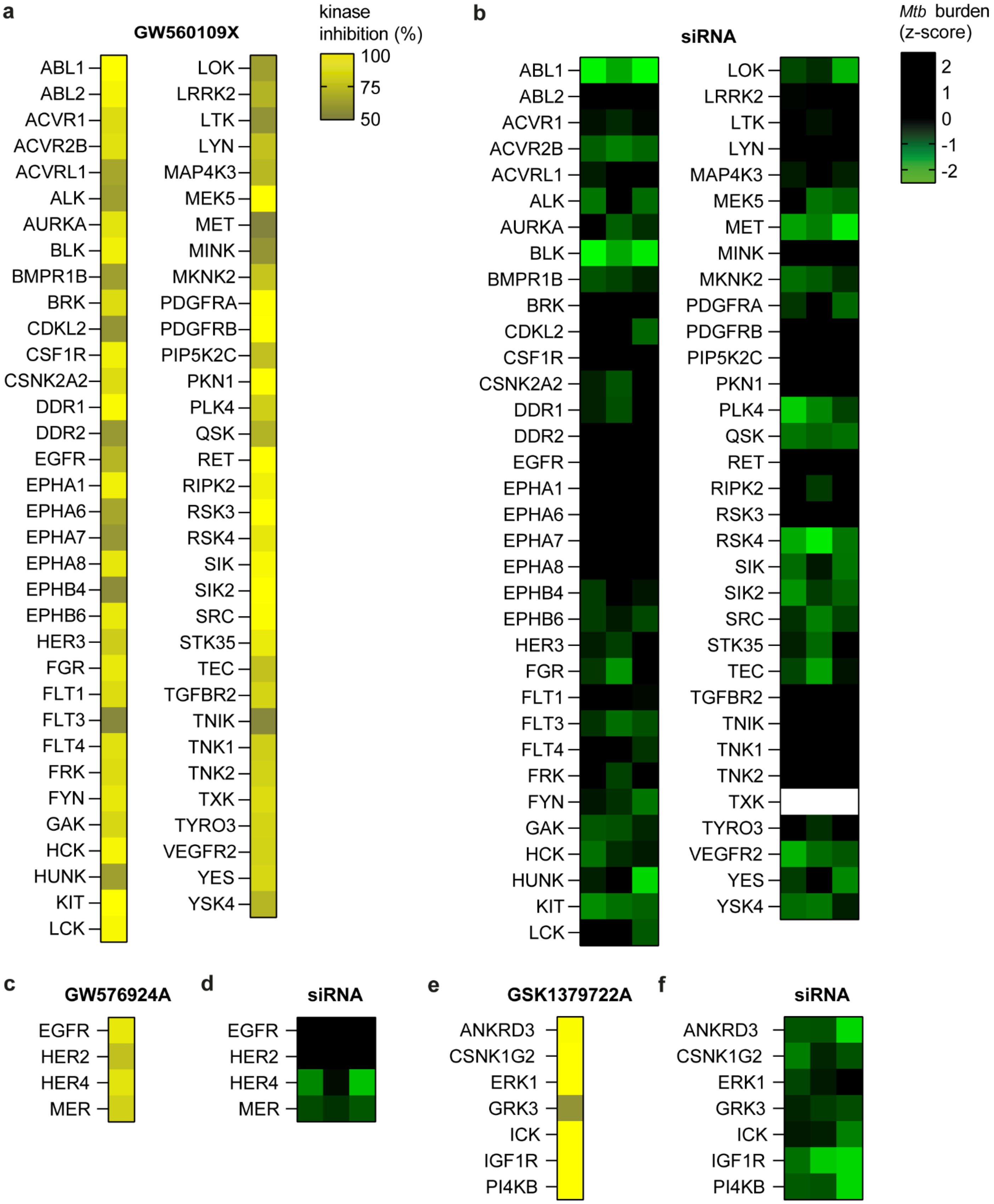

Fig. S5 Kinase inhibition profiles of *Mtb* hit compounds GW560109X, GW57924A and GSK1379722A. (**a**) Kinases inhibited >50% by GW560109X at 1 µM. (**b**) Effect of siRNA knockdown of the kinase targets depicted in (A) on the bacterial burden of *Mtb*-infected MelJuSo cells. (**c**) Kinases inhibited >50% by GW57924A at 1 µM. (**d**) Effect of siRNA knockdown of the kinase targets depicted in (c) on the *Mtb* bacterial burden. (**e**) Kinases inhibited >50% by GSK1379722A at 1 µM. (**f**) Effect of siRNA knockdown of the kinase targets depicted in (e) on the *Mtb* bacterial burden.

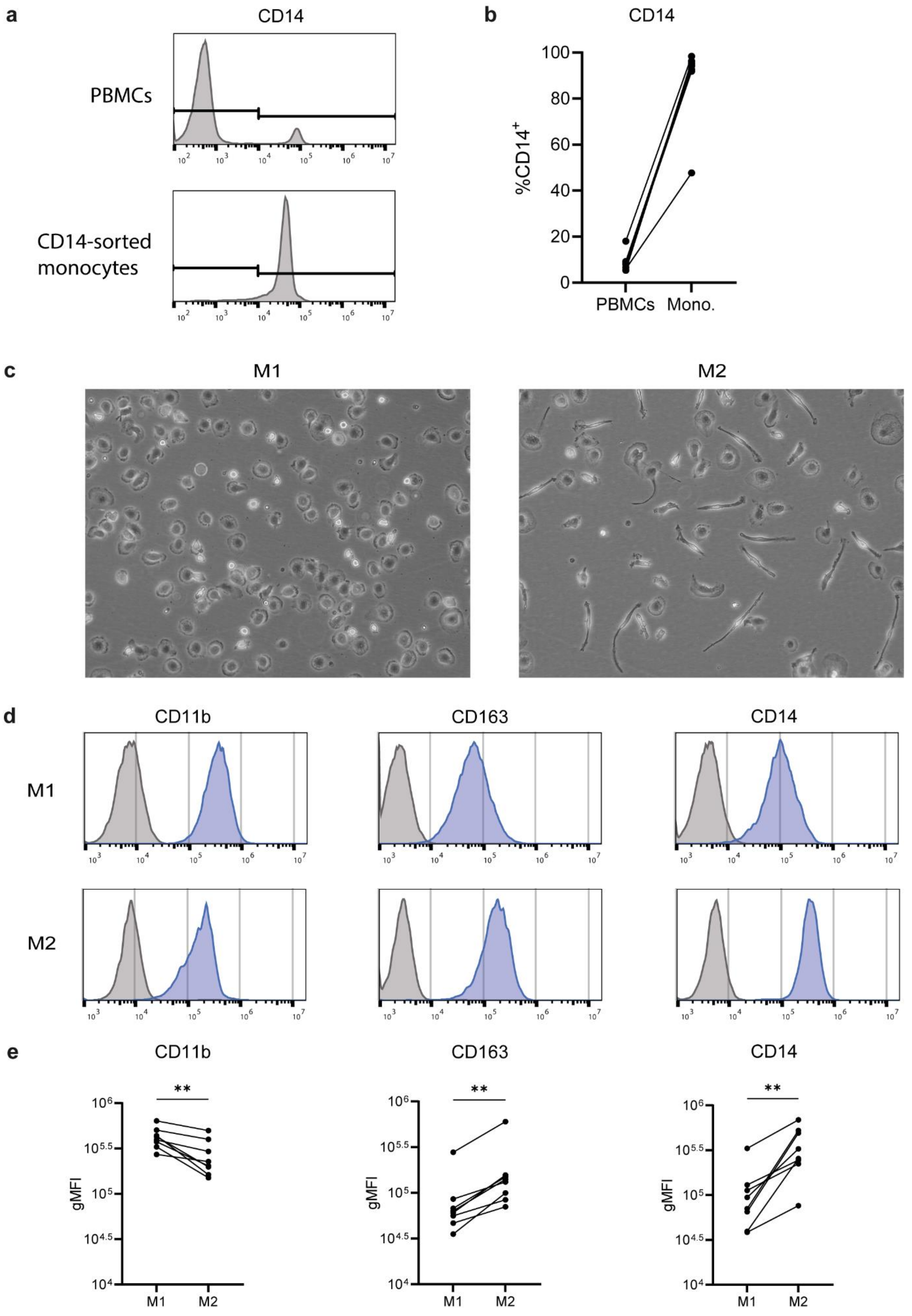

Fig. S6 Generation, morphology and phenotype of primary human macrophages. (**a**) Representative histograms showing CD14 expression in PBMCs and CD14-MACS sorted monocytes. (**b**) Enrichment of $CD14^+$ cells before and after MACS sorting. (**c**) Morphology of M1 and M2 macrophages. (**d**) Representative histograms showing distinct expression patterns of CD11b, CD163 and CD14 by M1 and M2 macrophages. (**e**) Expression of CD11b, CD163 and CD14 by M1 and M2 macrophages was tested for differences using Wilcoxon matched-paired signed rank tests. Statistically significant differences are shown by $^{**}p < 0.01$.
